# Reliable and Accurate CD4+ T Cell Count and Percent by the Portable Flow Cytometer CyFlow MiniPOC and “CD4 Easy Count Kit-Dry”, as Revealed by the Comparison with the Gold Standard Dual Platform Technology

**DOI:** 10.1371/journal.pone.0116848

**Published:** 2015-01-26

**Authors:** Milena Nasi, Sara De Biasi, Elena Bianchini, Lara Gibellini, Marcello Pinti, Tiziana Scacchetti, Tommaso Trenti, Vanni Borghi, Cristina Mussini, Andrea Cossarizza

**Affiliations:** 1 Department of Surgery, Medicine, Dentistry and Morphological Sciences, University of Modena and Reggio Emilia School of Medicine, via Campi 287, 41125 Modena, Italy; 2 Department of Clinical Pathology, BLU Laboratory, Nuovo Ospedale Civile Sant’Agostino Estense—NOCSAE, Baggiovara, Modena, Italy; 3 Infectious Diseases Clinics, Azienda Ospedaliero-Universitaria Policlinico, via del Pozzo 71, 41124 Modena, Italy; George Mason University, UNITED STATES

## Abstract

**Background:**

An accurate and affordable CD4+ T cells count is an essential tool in the fight against HIV/AIDS. Flow cytometry (FCM) is the “gold standard” for counting such cells, but this technique is expensive and requires sophisticated equipment, temperature-sensitive monoclonal antibodies (mAbs) and trained personnel. The lack of access to technical support and quality assurance programs thus limits the use of FCM in resource-constrained countries. We have tested the accuracy, the precision and the carry-over contamination of Partec CyFlow MiniPOC, a portable and economically affordable flow cytometer designed for CD4+ count and percentage, used along with the “CD4% Count Kit-Dry”.

**Materials and Methods:**

Venous blood from 59 adult HIV+ patients (age: 25–58 years; 43 males and 16 females) was collected and stained with the “MiniPOC CD4% Count Kit-Dry”. CD4+ count and percentage were then determined in triplicate by the CyFlow MiniPOC. In parallel, CD4 count was performed using mAbs and a CyFlow Counter, or by a dual platform system (from Beckman Coulter) based upon Cytomic FC500 (“Cytostat tetrachrome kit” for mAbs) and Coulter HmX Hematology Analyzer (for absolute cell count).

**Results:**

The accuracy of CyFlow MiniPOC against Cytomic FC500 showed a correlation coefficient (CC) of 0.98 and 0.97 for CD4+ count and percentage, respectively. The accuracy of CyFlow MiniPOC against CyFlow Counter showed a CC of 0.99 and 0.99 for CD4 T cell count and percentage, respectively. CyFlow MiniPOC showed an excellent repeatability: CD4+ cell count and percentage were analyzed on two instruments, with an intra-assay precision below ±5% deviation. Finally, there was no carry-over contamination for samples at all CD4 values, regardless of their position in the sequence of analysis.

**Conclusion:**

The cost-effective CyFlow MiniPOC produces rapid, reliable and accurate results that are fully comparable with those from highly expensive dual platform systems.

## Introduction

An estimated 35.3 (32.2–38.8) million people are living with human immunodeficiency virus (HIV) [1]. The epidemics continues to disproportionately affect sub-Saharan Africa, home to 70% of all new HIV infections in 2012 [1]. Since the virus kills, directly or indirectly, CD4+ T cells [2, 3], the accurate, reliable, and affordable CD4+ T cell count is essential in determining disease stage and progression [4]. An active monitoring of the immune system in both HIV patients and individuals who are regarded as “at-risk” is crucial in determining when antiretroviral therapy has to start, and in its monitoring [5, 6]. Moreover, although absolute CD4 counts are used for assessing the clinical status and eventual progression of the infection in adult patients, populations of lymphocytes including CD4+ are greater in children. Therefore, in HIV+ children it is more informative to measure the percentage of CD4+ T cells among the total lymphocyte population [7].

Since the beginning of the epidemics, flow cytometry (FCM) is clearly the “gold standard” for the enumeration of different lymphocyte populations and to follow different aspects of HIV+ patients [8–10], but typically this technique is very expensive and requires sophisticated equipment and trained personnel. In most cases, another expensive instrument, *i.e*., a hematology analyzer, is required for the absolute cell count. In addition, the lack of ready access to technical support and quality assurance programs limits the use of FCM in resource-constrained countries. In the last decade, several single flow platform instruments able to identify and count cells have been developed. These are mainly represented by flow cytometers that count the absolute CD4 cell number in a fixed volume, such as CyFlowSL BlueH, CyFlow GreenH, CyFlow CounterH (Partec GmbH, Münster, Germany), Guava EasyCD4 (Guava Technologies, Hayward, CA), Apogee Auto40 (Apogee Flow Systems, Hemel Hempstead, UK) [11]. These instruments are much less expensive and easier to perform than those based upon dual platform technology, or than those that require bead-assisted calibrations and reagents that require the cold chain.

More recently, it became clear that the fight against HIV/AIDS requires point-of-care (POC) technologies for rapid, reliable and affordable CD4+ analysis, to be used especially in rural areas [12]. These instruments are designed for minimal operator intervention; among them, there are a few modified flow cytometers such as the PointCare NOW (PointCare Technologies, Marlborough, MA, USA) and the CyFlow MiniPOC (Partec), or instruments that count cells utilizing dual-fluorescence image analysis such as Alere Pima CD4 (Alere Inc, Waltham, MA, USA) [13].

The CyFlow MiniPOC is a portable flow cytometer specifically designed for the automatic calculation of CD4+ T cell count and percentage. It is equipped with a 30 mW, 532 nm laser and three optical parameters for the detection of side scatter, orange and red fluorescence. It is a True Volumetric Absolute Counting instrument based on the precise counting and mechanical fluid volume measurement, that no need for reference sample or reference beads. It is remarkable that this instrument can be equipped with a rechargeable lithium battery dock or with solar panel. The CyFlow MiniPOC can be associated with a kit containing dry lyophilized reagents (the “CD4% Count Kit-Dry”) such as fluorochrome-conjugated monoclonal antibodies (mAbs), to avoid the cold chain.

Starting form whole blood, results concerning the CD4+ T cell count can be obtained in a relatively short time (typically, within 20 minutes from the venipuncture), so that the patient can receive this information before the visit by the clinician and the relative decision-making process. This has an extraordinary importance in rural areas. However, data are required that compare this system to those used for routine CD4+ T cell count by the more sophisticated standard of care systems.

In this study, we aimed to evaluate the accuracy of the CyFlow miniPOC instrument by comparing data obtained with this system, coupled with the “CD4% Count Kit-Dry” with those obtained by using two different reference systems suited for CD4 absolute and CD4 percent analysis on whole blood. Moreover, our study also aimed at evaluating the precision of the instrument, in particular in the presence of low CD4+ count and percentage, and the analysis of the effects of sample carry over contamination.

## Materials and Methods

### Patients and blood collection

The study was approved by the Institutional Review Board of the Dept. of Surgery, Medicine, Dentistry and Morphological Sciences of the University of Modena and Reggio Emilia, and has been performed in accordance with the ethical standards of the committee on human experimentation and the Helsinki Declaration. After written informed consent, a 3 mL sample of venous blood was collected in EDTA tubes from 59 HIV+ patients at the time of their routine visits for the CD4+ T cell count and quantification of plasma viral load. All patients were followed by the Infectious and Tropical Diseases Clinics of the University of Modena and Reggio Emilia (Northern Italy). The study population consisted of 59 HIV+ patients (16 females and 43 males) who were over 18 years of age (range 25–58 years). All but 2 were being treated with successful combination antiretroviral therapy.

### CD4+ T cell count and percentage analysis

Twenty µL of whole blood were stained with the “CD4% Count Kit-Dry” (Partec GmbH—a Sysmex Company, Münster, Germany) for the determination of CD4+ T cell count and percentage by the CyFlow MiniPOC instrument. In parallel, 20 µL of whole blood from the same patients were stained with the “CD4 Easy Count Kit” (for the determination of CD4+ T cell count) and the “CD4% Easy Count Kit” (for the determination of CD4+ T cell count and percentage) and analyzed by CyFlow Counter. All samples were stained and analyzed within 2 hours and all measures were performed in triplicate. Parallel blood samples were analyzed by the BLU Laboratory (Unified Laboratory of Baggiovara) that routinely counts CD4+ T cells by a dual platform system based upon Cytomics FC 500 (Cytostat tetrachrome kit, for the percentage of CD4+ cell) and Coulter HmX Hematology Analyzer (for the absolute lymphocyte count).

### CyFlow MiniPOC technical details

Following the preparation of EDTA blood specimen with Partec miniPOC CD4% Count Kit-dry according to the manufacturer’s instructions, the samples are inserted in the CyFlow miniPOC using a syringe. The CyFlow miniPOC sample port uses computer controlled stepper motor driven actuator so that the final volume injected in the system is accurately calculated by using the distance defined by the actuator and the specific diameter of the syringe. The main element of the CyFlow miniPOC Flow Cytometer is a flow cuvette where single blood cell stream is analysed. This is made of quartz glass, which contains a capillary with a diameter of 250 × 350 µm.

The fluidic system of the CyFlow miniPOC is used to transport blood cells from a three dimensional sample suspension to an orderly single cell stream passing through one illuminating laser beam. By regulating the air pressure the fluidic system ensures stable operation and it consists of a sheath fluid line and a sample line feeding into the flow cell. The CyFlow miniPOC is equipped with is equipped with 30 mW 532 nm laser and three optical parameters for the detection of side scattered light (SSC), orange (FL2) and red fluorescence (FL3). The side scattered light and the fluorescence light are collected at an angle of 90° degrees. The light is then subdivided into different wavelengths by optical filters. In the next step, the photomultiplier collect the different wavelengths by generating an electronic impulse. The instrument is triggered when this signal exceeds a predefined threshold level. The threshold is primarily used to reject non—cellular events such as debris or noise from optical and electronic sources. The data are displayed as 2-parameter dot plot and as digital numbers for both CD4 absolute count and CD4% values. Additional information are provided in Supplementary Material Section.

### Precision assessment

For the repeatability study we choose a whole blood sample with low CD4 absolute count, *i.e*. <300 CD4+ T cells/μl. The sample was stained 10 times individually according to the product data sheet of the “CD4% Count Kit—Dry” designed for the CyFlow MiniPOC. For bulk analysis, the sample was stained 12 times individually. After addition of Buffer 1, all 12 samples were pooled, carefully mixed and ten aliquots with a volume of 430 µL were transferred into CyFlow miniPOC tubes. Buffer 2 was added right before analysis of the stained blood samples as described in the Operating Manual.

### Carry-over contamination analysis

For the carry-over study, 3 whole blood samples were chosen which differed significantly in their CD4 count value: low CD4 count, defined as <300 CD4+ T cells/μL, medium CD4 count, defined as 300–600 CD4+ T cells/μL and high CD4 count, defined as >600 CD4+ T cells/μL. Whole blood samples were stained according to the product data sheet of the CyFlow miniPOC CD4% Count Dry Kit and the analyses were performed in triplicate with the subsequent order: medium—high—low—medium—medium—low—low—high—high—medium.

### Statistical analysis

The data obtained by test and reference system were compared using correlation coefficient and linear regression analysis. The CD4+ T-cell count (or CD4% value) obtained by the reference system was plotted on the x-axis against the CD4+ T-cell count (or CD4% value) obtained by the test system on the y-axis. Bland-Altman plots were used to evaluate the agreement between two methods by plotting the mean of the values on the x-axis and the difference of the values on the y-axis [14]. Precision expressed as the coefficient of variation (CV) was determined by dividing the standard deviation (SD) of the 10 measurements by the mean (%CV = SDx100/mean). Non parametric Mann-Whitney test was used to compare values from the same sample in the setting of the carry-over contamination analysis. All the above-mentioned statistical analyses were performed by Prism 5.0 (GraphPad, La Jolla, CA) software.

## Results

### Comparison between CyFlow MiniPOC and CyFlow Counter

**Figure 1:**
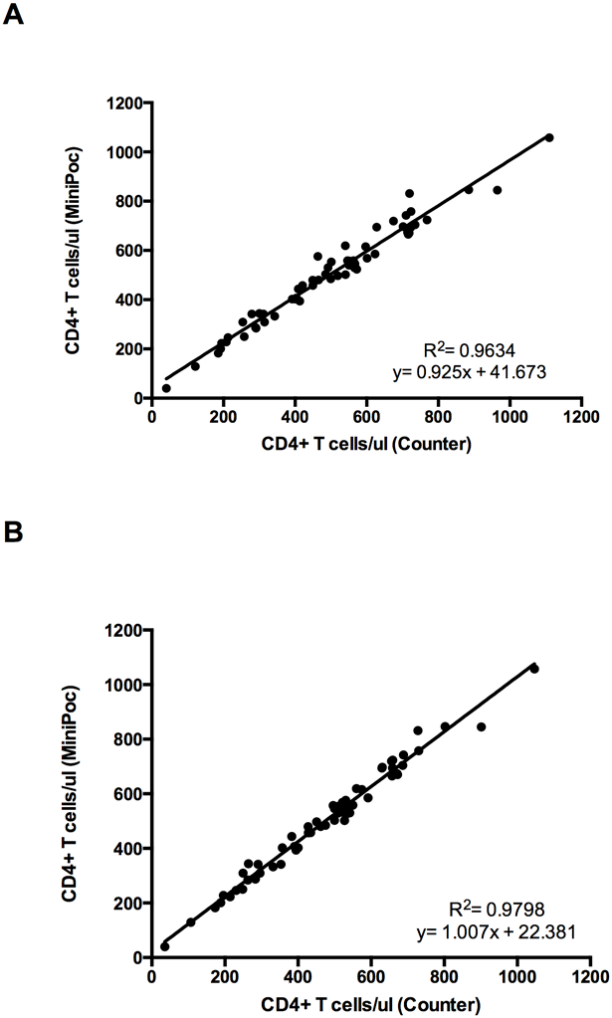
Comparison of the CyFlow MiniPOC and CyFlow Counter. Correlation and R^2^ values for CD4+ T cell counts obtained by Cyflow MiniPOC and CyFlow Counter by using the CD4 easy count kit (A) and the CD4% easy count kit (B).

**Figure 2:**
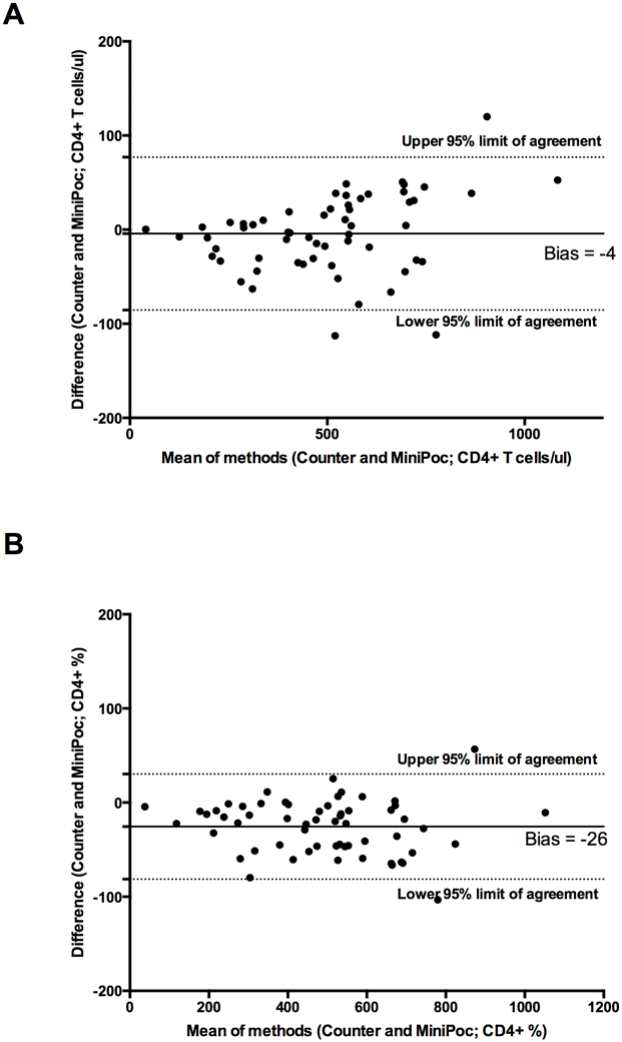
Comparison of the CyFlow MiniPOC and CyFlow Counter. Bland-Altman plot comparing absolute CD4+ T cell counts obtained by Cyflow MiniPOC and CyFlow Counter by using the CD4 easy count kit (A) and the CD4% easy count kit (B).

**Figure 3:**
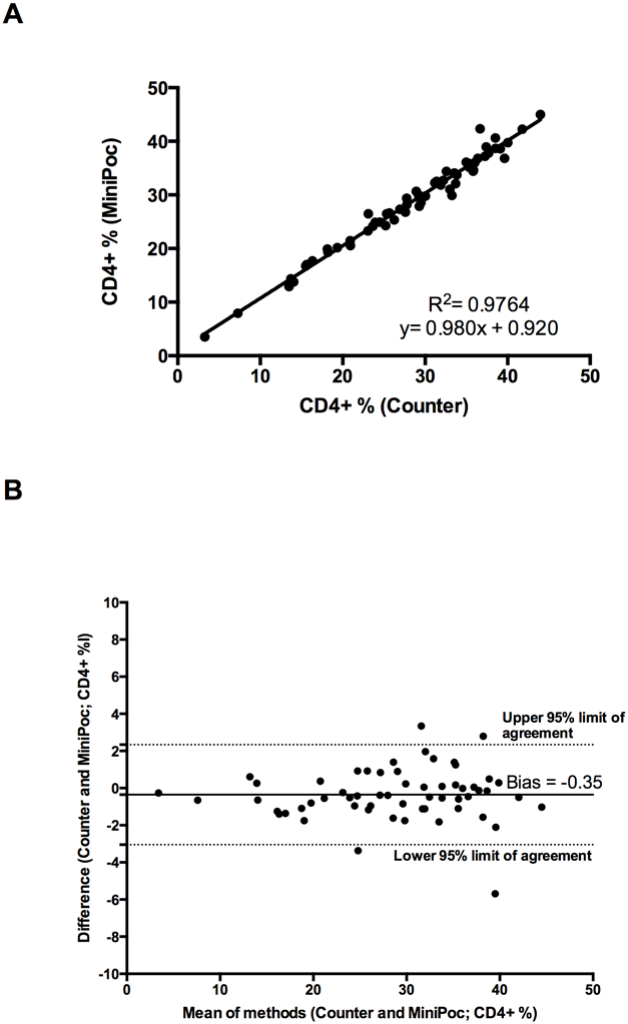
Comparison of the CyFlow MiniPOC and CyFlow Counter. Linear Regression(A) and Bland-Altman Plot (B) for CD4+ T cell percent obtained by Cyflow MiniPOC and CyFlow Counter by using the CD4% easy count kit.

The values of CD4+ T cell count obtained by CyFlow MiniPOC showed a very good correlation with those obtained with the CyFlow Counter either by using the “CD4 Easy Count Kit” (Fig. 1A; R^2^ = 0.963, correlation coefficient 0.98) or by using the “CD4% Easy Count Kit” (Fig. 1B; R^2^ = 0.979, correlation coefficient 0.99). The Bland-Altman plot indicates that the overall, absolute CD4+ T cell counts obtained by the two methods were in excellent agreement (Fig. 2A and 2B). The values of CD4+ T cell percent obtained by CyFlow MiniPOC showed a strong correlation with those obtained with the CyFlow Counter by using the CD4% easy count kit (Fig. 3A; R^2^ = 0.976, correlation coefficient 0.99). All individual data points represented in Figs. 1–3 are shown in S1 Table.

### Comparison between CyFlow MiniPOC and dual platform Coulter FC500

**Figure 4:**
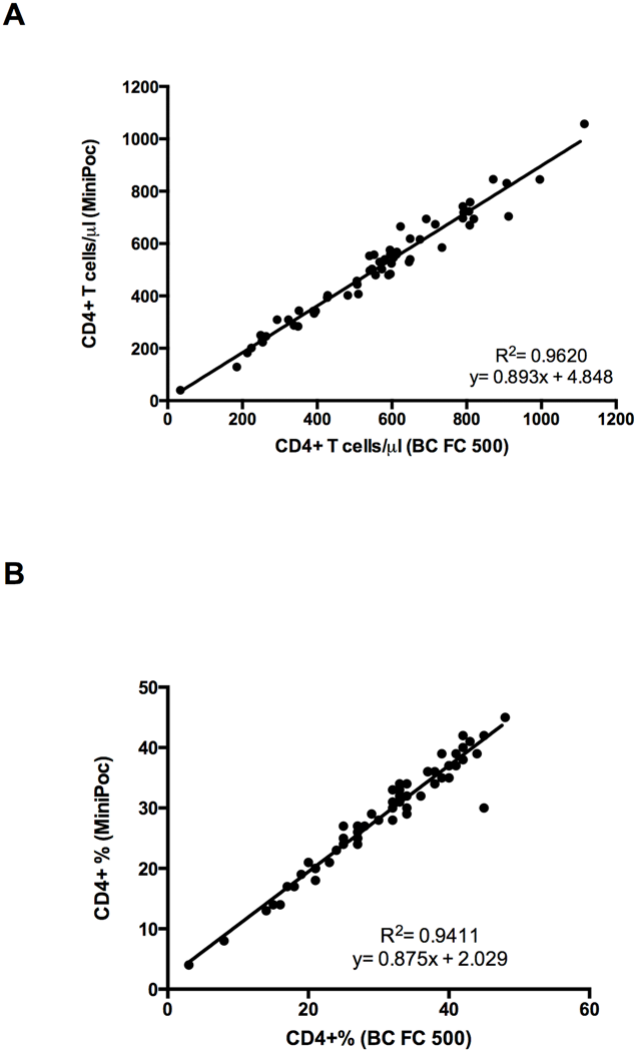
Comparison of the CyFlow MiniPOC and BC FC 500. Correlation and R^2^ values for CD4+ T cell count (A) and percentage (B) obtained by Cyflow MiniPOC and BC FC 500 reference system.

**Figure 5:**
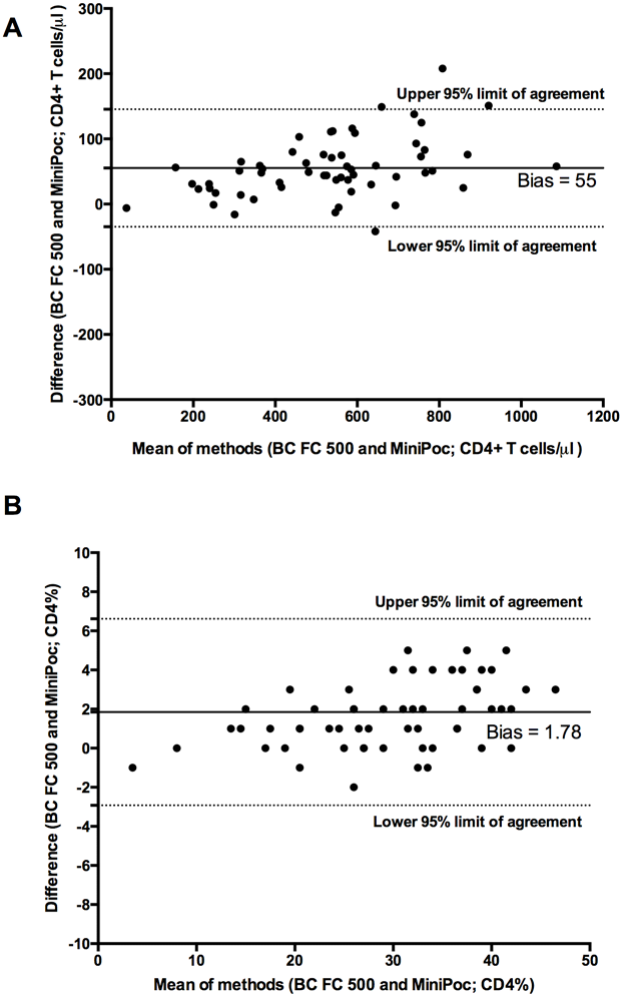
Comparison of the CyFlow MiniPOC and BC FC 500. Bland-Altman plot comparing absolute CD4+ T cell count (A) and percentage (B) obtained by Cyflow MiniPOC and BC FC 500 reference system

The data obtained by CyFlow MiniPOC display a good correlation with those obtained with the Coulter FC500 either considering CD4+ T cell count (Fig. 4A; R^2^ = 0.962, correlation coefficient 0.98) either considering CD4+ T cell percentage (Fig. 4B; R^2^ = 0.941, correlation coefficient 0.97). The Bland-Altman plot shows that in both case the two methods were in close agreement (Fig. 5A and 5B). All individual data points represented in Figs. 4 and 5 are shown in S2 Table.

### Precision assessment

**Figure 6:**
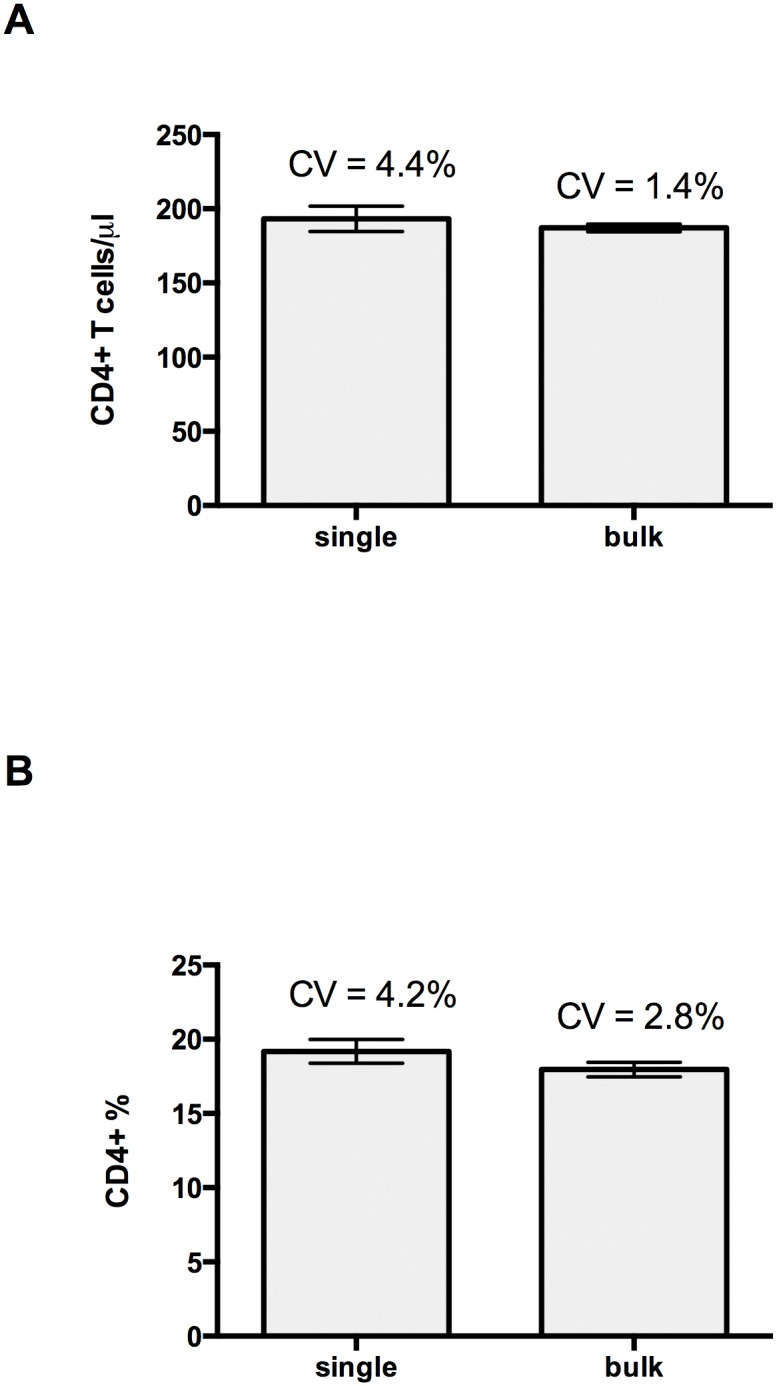
Precision assessment. Bar charts with error bars indicating the standard deviation of CD4 T cell count (A) and CD4% (B) of a sample analyzed 10 times in single or in bulk. CV = Coefficient of variation.

The mean and the coefficient of variation (CV) of CD4 absolute count, obtained with the CyFlow MiniPOC, was 193 CD4+ T cells/μL and 4.4% for the sample analyzed in single mode, and 187 CD4+ T cells/μL and 1.4% for the sample analyzed in bulk (Fig. 6A). The mean and the CV of CD4% was 19% and 4.2%, respectively, for the sample analyzed in single mode, and 18% and 2.8% for the sample analyzed in bulk (Fig. 6B). All individual data points represented in Fig. 6 are shown in S3 Table.

### Carry-over contamination analysis

**Figure 7:**
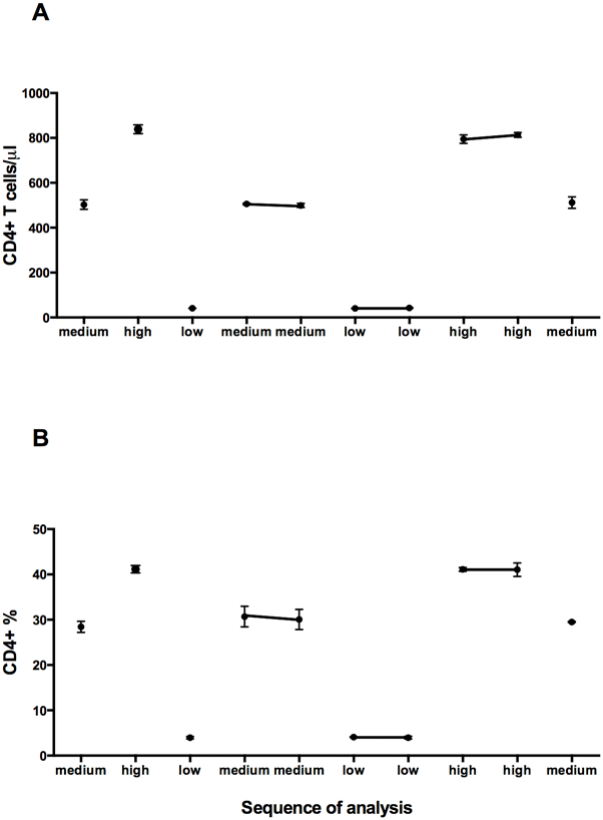
Carry-over contamination analysis. The CD4 absolute count (A) and CD4% (B) were plotted as the mean value of three analysis sequences. Error bars indicate the standard deviation of three measurements.

As shown in Fig. 7, using the CyFlow MiniPOC, we observed no significant differences among all samples with low CD4 count and percentage, all those with medium CD4 count and percentage and all those with high CD4 count and percentage, regardless of their position in the sequence of analysis. All individual data points represented in Fig. 7 are shown in S4 Table.

## Discussion

Highly effective antiretroviral therapy has dramatically decreased the mortality of HIV infection and significantly prolonged the survival of patients [15, 16], who now have a life expectancy very close to that of non-infected individuals [17]. However, either the initiation of therapy or the analysis of its efficacy depend on the evaluation of laboratory parameters such as CD4+ T cell count, and indeed a strong debate exist that concerns, for example, the number of CD4+ T cells at which therapy has to start [18]. As a consequence, measuring such cells, along with the plasma viral load, is absolutely crucial and has a pivotal importance for an optimal management of HIV+ patients.

More than 95% of infections occur in low and middle-income countries, where the number of laboratories that can use sophisticated molecular biology techniques is extremely low. Thus, even if nowadays molecular virology has fantastic technological possibilities, the quantification of plasma viral load is still far from being a test that has a large use. Thus, the simple (and old) CD4+ T cell count remains the main option to adequately follow a HIV+ patient in resource-constrained countries [19].

Currently, the gold standard for CD4+ T cell count is a test that requires at least 3 or 4 (if not more) mAbs conjugated with different fluorochromes, whose binding to peripheral blood cells is analyzed on a flow cytometer often equipped with 2 lasers. For several years, different groups, including ours, have largely used multilaser and polychromatic flow cytometry to deeply investigate the immune system during HIV infection [20–24]. Unfortunately, the global cost of similar instruments, their maintenance and the required reagents cannot realistically be afforded outside clinics or hospitals with medium to high budgets. Moreover, flow cytometers that provide excellent measurements along with several additional immunological information (*e.g*. the number of CD8+ T cells or T cell activation), are not transportable, nor they can be used as core instruments where skilled operators able to run, maintain and repair, as well as trained personnel able to interpret complex data, are not present.

Most HIV+ patients live far from hospital, and do not have the possibility to travel to distant clinics—or they cannot do this for social reasons, *i.e*. they do not want to show that they have to go regularly to the hospital for CD4+ T cell count, that means that they have HIV infection. Thus, the need for a reliable and economic point of care (POC) diagnostic for the count of CD4+ T cells is a fundamental tool in the fight against HIV/AIDS. Recently, a panel of WHO experts have prepared guidelines for the use of POC diagnostics in resource limited environments, that go under the acronym of ASSURED. The criteria that were identified are related to the fact that every tool has to be economically Affordable, Sensitive, Specific, User-friendly, Robust and rapid, Equipment-free, Deliverable [25]. Furthermore, for obvious reasons linked to the logistics and weather temperatures, it is crucial that the reagents, and in particular fluorochrome-conjugated mAbs, do not require the cold chain.

**Figure 8:**
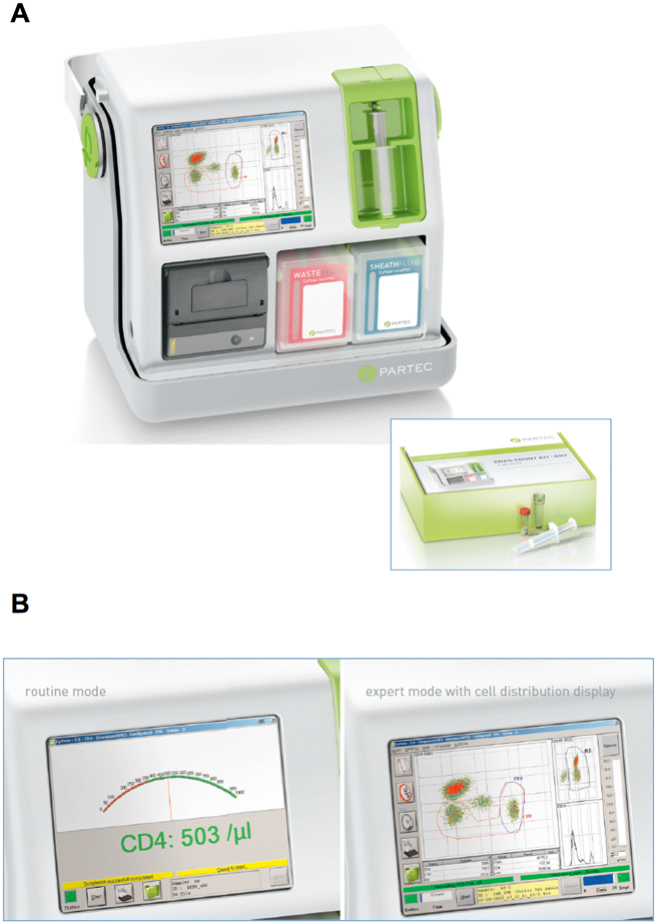
Partec CyFlow MiniPOC. The Partec CyFlow MiniPOC counting device (A) and its display (B) in routine (left) or expert mode (right). Both images are used with permission of Partec/Sysmex GmbH, Germany.

The main aim of our study was to compare a system that fulfills the ASSURED criteria, such as that formed by the CyFlow MiniPOC and the CD4% Count Dry-Kit (shown in Fig. 8A), with the gold standard, dual platform system that is routinely used in an Italian Hospital since several years, and with another single-platform low cost system, *i.e*. the CyFlow Counter. We found that the results obtained with the CyFlow miniPOC with the use of the CD4% count dry kit are comparable either to those obtained with an independent reference system, i.e. the Beckman Coulter FC500 plus Coulter HmX, or to the single platform CyFlow Counter. Furthermore, the results obtained with the dry kits designed for the CyFlow miniPOC were fully comparable with the liquid kits “CD4 easy count kit” and “CD4% easy count kit” used with the CyFlow Counter.

In our hands, the CyFlow miniPOC showed an excellent repeatability using whole blood samples with low CD4 absolute and CD4 percent values, and indeed the assay precision was below +5% deviation. Finally, the CyFlow miniPOC showed no carry-over contamination among samples. Thus, the cost-effective and portable instrument CyFlow MiniPOC is able to produce rapid, reliable and accurate results that are fully comparable with highly expensive dual platform systems, and allows to analyze a large number of samples per day.

As far as HIV+ adults are concerned, the guidelines indicate to consider the absolute number of CD4+ T cells to monitor the infection. Here we show that this system is extremely reliable and provides the same results obtained with instruments that are extremely more expensive and complex. In HIV+ children, it is important to measure also the percentage of CD4+ lymphocytes. The assay we used is based upon the staining of whole blood with both anti-CD4 and anti-CD45 mAbs. CD45 is expressed on the plasma membrane of all leukocytes, but with a different intensity, that depends on the cell type. Thus, lymphocytes can be easily recognized on the bases of the side scatter and CD45 expression can be electronically gated, and the percentage of those expressing CD4 can be easily calculated. Such operation can be run either in “expert mode”, where the operator can decide what to do, or in “routine mode”, where the gates are fixed and there is no need of any expert intervention (see Fig. 8B).

A last but not least consideration is finally required. The quality of CD4+ T cell count obtained by the system we have tested is fully comparable to the current standard of care. Thus, due to the continuous cuts of budgets that we are experiencing, an accurate and extremely low cost assay for CD4+ T cell count can be used also by sophisticated laboratories in resource “for the moment not so poor” settings.

## Supporting Information

S1 Document Additional technical information concerning the staining and the CyFlow MiniPOC. (DOCX)

S2 Document Authorization from Partec Sysmex to publish Fig 8. (PDF)

S1 Table Comparison between CyFlow MiniPOC and CyFlow Counter. Individual data points used for Figs. 1–3.
(DOCX)

S2 Table Comparison between CyFlow MiniPOC and BC FC 500. Individual data points used for Figs. 4 and 5.
(DOCX)

S3 Table Precision assessment. Individual data points used for Fig. 6.
(DOCX)

S4 Table Carry-over contamination analysis. Individual data points used for Fig. 7.
(DOCX)
