# Supplementary material for: Reliable and Accurate CD4+ T Cell Count and Percent by the Portable Flow Cytometer CyFlow MiniPOC and “CD4 Easy Count Kit-Dry”, as Revealed by the Comparison with the Gold Standard Dual Platform Technology
Source: PLoS One. 2015 Jan 26;10(1):e0116848. doi: 10.1371/journal.pone.0116848 (PMC4306486; doi:10.1371/journal.pone.0116848)
Supplement: S1 Document — (DOCX) [file pone.0116848.s001.docx]

**METHODOLOGY AND ADDITIONAL TECHNICAL INFORMATION**

Blood specimen anticoagulated with EDTA are stained by using with Partec miniPOC CD4% Count Kit-dry (see: http://www.sysmex-partec.com/fileadmin/media/pdf/PARTEC_PEH-FactSheet-042013.pdf).

Then, samples are inserted in the CyFlow® miniPOC using a syringe. The CyFlow® miniPOC sample port uses computer controlled stepper motor driven actuator so that the final volume injected in the system is accurately calculated by using the distance defined by the actuator and the specific diameter of the syringe. The main element of the CyFlow^®^ miniPOC Flow Cytometer is a flow cuvette where single blood cell stream is analysed. This is made of quartz glass, which contains a capillary with a diameter of 250 x 350 µm.

The fluidic system of the CyFlow^®^ miniPOC is used to transport blood cells from a three dimensional sample suspension to an orderly single cell stream passing through one illuminating laser beam. By regulating the air pressure the fluidic system ensures stable operation and it consists of a sheath fluid line and a sample line feeding into the flow cell. The CyFlow® miniPOC is equipped with 30 mW 532 nm laser and three optical parameters for the detection of side scattered light (SSC), orange (FL2) and red fluorescence (FL3). The side scattered light and the fluorescence light are collected at an angle of 90° degrees. The light is then subdivided into different wavelengths by optical filters.

In the next step, the photomultiplier collect the different wavelengths by generating an electronic impulse. The instrument is triggered when this signal exceeds a predefined threshold level. The threshold is primarily used to reject non - cellular events such as debris or noise from optical and electronic sources.

The data are displayed as 2-parameter dot plot and as digital numbers for both CD4 absolute count and CD4% values.

Other technical specifications are:

**Size** W 268 mm x H 243 mm x D 186 mm

**Weight** 6.5 kg

**Power Supply** Input voltage 100-240 VAC
 50/60 Hz
Output voltage 12 VDC
Output current 5.5 A

**Maximum sound power level** < 70 dBA

**Installation/overvoltage category** 3/III

**Degree of protection** IP 20

**Operating Environment** Temperature 15-30°C
Humidity 20-85% relative (non-condensing)

**Applications** HIV/AIDS Patient Monitoring, Immunophenotyping, 2 Colour Analysis and Determination of Particle Concentrations with TVAC.

**TVAC** True Volumetric Absolute Counting.Based on the precise counting and mechanical fluid volume measurement. No need for reference sample or reference beads.

**Instrument Check** Count Check Beads green – dry (included in Partec miniPOC CD4% count kit – dry (Partec Code No. 05-8409-d))

**Setup Time** Max. 5 minutes

**Parameters** 3 optical parameters: SSC, FL2, FL3

**Particle Size Range** 0.5 – 50 µm (standard cuvette)

**Maximum Acquisition Speed** 15,000 events/sec

**Acquisition Stop Time** volume-based

**Trigger** FL2 (CD4^+^ absolute count, Count Check Beads green)
FL3 (CD4% count)

**Data Resolution** 65,536 (16 bit)

**Graphics Resolution** 256 channels resolution for 1P histogram
256/256 channels resolution for 2P dot plot

**Service** Please contact your local supplier or Partec (supportcenter@partec.com)

**Warranty** 12 months on all parts except filters, mirrors, other quartz or glass part, and disposables as long as not otherwise stated

**2. CyFlow^®^ miniPOC Optics**

**Laser/Output** Green NdYAG: 30 mW at 532 nm

**Detectors** 3 (SSC, FL2, FL3)

**Filters** Standard setup and filters for SSC, FL2 and FL3

**Excitation Optics** Elliptical 15 µm x 100 µm at 488 nm

**3. CyFlow^®^ miniPOC Fluidics**

**Flow Cuvette** Synthetic quartz flow cuvette with centric flow channel (250 x 350 µm) for laminar sample transport with Sheath Fluid for scatter and fluorescence light detection.

**Sample Delivery** Computer controlled precision syringe pump for contamination-free sample transport. Built-in vacuum pump (200 mbar) for Sheath Fluid and sample transport.

**Sampling Volume** Continuous up to 1200 µl
At least 840 µl for True Volumetric Absolute Counting (TVAC)

**Flow Rates** Sample volume speed adjustable continuously between 0.2 and 20 µl/sec

**Fluidics Volume** 2 x container for Sheath Fluid and Waste (270 ml)

**4. CyFlow^®^ miniPOC Electronics and Computer**

**Electronics** Signal processing
Amplifiers
16 bit ADC
Trigger

**Computer** Built-in Computer
 Intel Celeron computer processor
 5.7’’ TFT colour touchscreen (SVGA)
 Keyboard (optional)
 Mouse (optional)
 Printer (built-in)

**5. CyView Software**

**Software** Windows™ Software
 FCS 2.0 standard list mode format
 Real-time acquisition
 Report (automatic calculation)

**Acquisition Gating** Lower level hardware thresholds for event triggering parameters, adjusted by software
Region (up to 16 spots)
Range

**Gating** On- and offline gating, gating stored with Script

**Script** Saves Layout of the Measurement (Regions, Scalings), Gain and speed values as default.

**Report** Automatic calculation of CD4 and CD4%
