# Supplementary material for: Reliable and Accurate CD4+ T Cell Count and Percent by the Portable Flow Cytometer CyFlow MiniPOC and “CD4 Easy Count Kit-Dry”, as Revealed by the Comparison with the Gold Standard Dual Platform Technology
Source: PLoS One. 2015 Jan 26;10(1):e0116848. doi: 10.1371/journal.pone.0116848 (PMC4306486; doi:10.1371/journal.pone.0116848)
Supplement: S1 Table — Individual data points used for Figs. 1–3. (DOCX) [file pone.0116848.s003.docx]

**Table S1: Comparison between CyFlow MiniPOC and CyFlow Counter**

|  | **CyFlow Counter** |  |  |  | **CyFlow® MiniPOC** | |
| --- | --- | --- | --- | --- | --- | --- |
|  | **CD4 easy count kit** | **CD4% easy count kit** |  |  | **Partec miniPOC CD4% count kit - dry** | |
| **ID sample** | **CD4 + T cells/ul** | **CD4 + T cells/ul** | **CD4%** |  | **CD4 count** | **CD4%** |
| 001_1 | 554,00 | 488,00 | 35,62 |  | 555,00 | 42,75 |
| 001_2 | 564,00 | 539,00 | 37,65 |  | 558,00 | 42,00 |
| 001_3 | 539,00 | 461,00 | 36,68 |  | 559,00 | 42,27 |
| AVERAGE OF THE 3 SAMPLES | 552,33 | 496,00 | 36,65 |  | 557,33 | 42,34 |
| 002_1 | 744,00 | 696,00 | 32,60 |  | 686,00 | 31,36 |
| 002_2 | 673,00 | 608,00 | 31,47 |  | 690,00 | 33,79 |
| 002_3 | 687,00 | 588,00 | 32,58 |  | 715,00 | 32,95 |
| AVERAGE | 701,33 | 630,67 | 32,22 |  | 697,00 | 32,70 |
| 003_1 | 428,00 | 417,00 | 37,58 |  | 397,00 | 37,30 |
| 003_2 | 390,00 | 430,00 | 38,76 |  | 406,00 | 39,84 |
| 003_3 | 381,00 | 354,00 | 36,74 |  | 404,00 | 36,33 |
| AVERAGE | 399,67 | 400,33 | 37,69 |  | 402,33 | 37,82 |
| 004_1 | 580,00 | 552,00 | 36,70 |  | 564,00 | 37,72 |
| 004_2 | 534,00 | 568,00 | 38,75 |  | 570,00 | 40,13 |
| 004_3 | 526,00 | 530,00 | 36,75 |  | 542,00 | 39,07 |
| AVERAGE | 546,67 | 550,00 | 37,40 |  | 558,67 | 38,97 |
| 005_1 | 530,00 | 516,00 | 15,82 |  | 574,00 | 17,69 |
| 005_2 | 479,00 | 546,00 | 15,52 |  | 539,00 | 17,23 |
| 005_3 | 495,00 | 465,00 | 15,56 |  | 547,00 | 16,17 |
| AVERAGE | 501,33 | 509,00 | 15,63 |  | 553,33 | 17,03 |
| 006_1 | 418,00 | 424,00 | 29,80 |  | 483,00 | 27,40 |
| 006_2 | 412,00 | 429,00 | 29,30 |  | 439,00 | 27,61 |
| 006_3 | 432,00 | 432,00 | 28,78 |  | 450,00 | 28,66 |
| AVERAGE | 420,67 | 428,33 | 29,29 |  | 457,33 | 27,89 |
| 007_1 | 496,00 | 538,00 | 32,75 |  | 521,00 | 30,77 |
| 007_2 | 496,00 | 543,00 | 33,18 |  | 540,00 | 31,32 |
| 007_3 | 484,00 | 543,00 | 33,05 |  | 530,00 | 30,99 |
| AVERAGE | 492,00 | 541,33 | 32,99 |  | 530,33 | 31,03 |
| 008_1 | 188,00 | 186,00 | 16,99 |  | 189,00 | 18,14 |
| 008_2 | 172,00 | 174,00 | 16,94 |  | 180,00 | 17,46 |
| 008_3 | 195,00 | 159,00 | 15,07 |  | 178,00 | 17,47 |
| AVERAGE | 185,00 | 173,00 | 16,33 |  | 182,33 | 17,69 |
| 009_1 | 597,00 | 565,00 | 36,15 |  | 574,00 | 36,33 |
| 009_2 | 613,00 | 521,00 | 36,18 |  | 580,00 | 36,27 |
| 009_3 | 593,00 | 478,00 | 35,70 |  | 550,00 | 35,48 |
| AVERAGE | 601,00 | 521,33 | 36,01 |  | 568,00 | 36,03 |
| 010_1 | 301,00 | 291,00 | 25,89 |  | 307,00 | 25,28 |
| 010_2 | 316,00 | 306,00 | 26,56 |  | 299,00 | 25,08 |
| 010_3 | 326,00 | 290,00 | 26,32 |  | 321,00 | 25,65 |
| AVERAGE | 314,33 | 295,67 | 26,26 |  | 309,00 | 25,34 |
| 011_1 | 776,00 | 688,00 | 23,55 |  | 766,00 | 23,44 |
| 011_2 | 751,00 | 635,00 | 22,79 |  | 719,00 | 23,49 |
| 011_3 | 779,00 | 654,00 | 22,82 |  | 685,00 | 22,94 |
| AVERAGE | 768,67 | 659,00 | 23,05 |  | 723,33 | 23,29 |
| 012_1 | 634,00 | 629,00 | 38,47 |  | 694,00 | 40,37 |
| 012_2 | 639,00 | 633,00 | 39,39 |  | 679,00 | 40,23 |
| 012_3 | 611,00 | 627,00 | 37,67 |  | 710,00 | 41,25 |
| AVERAGE | 628,00 | 629,67 | 38,51 |  | 694,33 | 40,62 |
| 013_1 | 261,00 | 300,00 | 7,06 |  | 335,00 | 7,65 |
| 013_2 | 324,00 | 299,00 | 7,38 |  | 347,00 | 8,12 |
| 013_3 | 350,00 | 273,00 | 7,40 |  | 344,00 | 8,02 |
| AVERAGE | 311,67 | 290,67 | 7,28 |  | 342,00 | 7,93 |
| 014_1 | 734,00 | 654,00 | 17,78 |  | 761,00 | 21,04 |
| 014_2 | 753,00 | 704,00 | 19,82 |  | 731,00 | 19,83 |
| 014_3 | 642,00 | 708,00 | 20,49 |  | 734,00 | 19,62 |
| AVERAGE | 709,67 | 688,67 | 19,36 |  | 742,00 | 20,16 |
| 015_1 | 214,00 | 188,00 | 17,05 |  | 227,00 | 20,80 |
| 015_2 | 185,00 | 202,00 | 18,51 |  | 235,00 | 19,46 |
| 015_3 | 224,00 | 197,00 | 18,87 |  | 222,00 | 19,41 |
| AVERAGE | 207,67 | 195,67 | 18,14 |  | 228,00 | 19,89 |
| 016_1 | 414,00 | 403,00 | 27,71 |  | 448,00 | 27,81 |
| 016-2 | 396,00 | 367,00 | 27,63 |  | 432,00 | 27,51 |
| 016_3 | 416,00 | 379,00 | 28,03 |  | 451,00 | 29,22 |
| AVERAGE | 408,67 | 383,00 | 27,79 |  | 443,67 | 28,18 |
| 017_1 | 446,00 | 412,00 | 25,45 |  | 470,00 | 26,91 |
| 017_2 | 472,00 | 496,00 | 25,92 |  | 483,00 | 27,22 |
| 017_3 | 479,00 | 478,00 | 25,61 |  | 488,00 | 25,74 |
| AVERAGE | 465,67 | 462,00 | 25,66 |  | 480,33 | 26,62 |
| 018_1 | 42,00 | 38,00 | 3,49 |  | 41,00 | 3,64 |
| 018_2 | 39,00 | 35,00 | 3,21 |  | 39,00 | 3,28 |
| 018_3 | 40,00 | 34,00 | 3,11 |  | 40,00 | 3,68 |
| AVERAGE | 40,33 | 35,67 | 3,27 |  | 40,00 | 3,53 |
| 019_1 | 701,00 | 668,00 | 43,83 |  | 732,00 | 45,33 |
| 019_2 | 670,00 | 649,00 | 43,62 |  | 714,00 | 44,99 |
| 019_3 | 653,00 | 651,00 | 44,52 |  | 712,00 | 44,72 |
| AVERAGE | 674,67 | 656,00 | 43,99 |  | 719,33 | 45,01 |
| 020_1 | 729,00 | 763,00 | 30,29 |  | 763,00 | 30,81 |
| 020_2 | 710,00 | 742,00 | 30,83 |  | 744,00 | 28,79 |
| 020_3 | 732,00 | 685,00 | 28,97 |  | 766,00 | 29,81 |
| AVERAGE | 723,67 | 730,00 | 30,03 |  | 757,67 | 29,80 |
| 021_1 | 744,00 | 753,00 | 42,42 |  | 828,00 | 44,38 |
| 021_2 | 714,00 | 720,00 | 41,90 |  | 834,00 | 41,21 |
| 021_3 | 701,00 | 711,00 | 41,01 |  | 832,00 | 41,26 |
| AVERAGE | 719,67 | 728,00 | 41,78 |  | 831,33 | 42,28 |
| 022_1 | 564,00 | 510,00 | 30,91 |  | 511,00 | 31,87 |
| 022_2 | 442,00 | 495,00 | 31,42 |  | 509,00 | 36,17 |
| 022_3 | 450,00 | 494,00 | 31,16 |  | 489,00 | 28,81 |
| AVERAGE | 485,33 | 499,67 | 31,16 |  | 503,00 | 32,28 |
| 023_1 | 980,00 | 844,00 | 37,06 |  | 832,00 | 37,36 |
| 023_2 | 956,00 | 914,00 | 39,95 |  | 807,00 | 39,43 |
| 023_3 | 958,00 | 946,00 | 40,32 |  | 895,00 | 39,07 |
| AVERAGE | 964,67 | 901,33 | 39,11 |  | 844,67 | 38,62 |
| 024_1 | 468,00 | 517,00 | 36,16 |  | 587,00 | 35,39 |
| 024_2 | 467,00 | 521,00 | 35,90 |  | 587,00 | 35,22 |
| 024_3 | 455,00 | 553,00 | 33,95 |  | 554,00 | 34,89 |
| AVERAGE | 463,33 | 530,33 | 35,34 |  | 576,00 | 35,17 |
| 025_1 | 266,00 | 353,00 | 29,60 |  | 341,00 | 28,43 |
| 025_2 | 346,00 | 351,00 | 29,26 |  | 339,00 | 28,11 |
| 025_3 | 225,00 | 356,00 | 29,57 |  | 346,00 | 29,20 |
| AVERAGE | 279,00 | 353,33 | 29,48 |  | 342,00 | 28,58 |
| 026_1 | 392,00 | 410,00 | 21,49 |  | 402,00 | 22,30 |
| 026_2 | 426,00 | 371,00 | 21,05 |  | 386,00 | 21,42 |
| 026_3 | 421,00 | 402,00 | 20,17 |  | 394,00 | 20,64 |
| AVERAGE | 413,00 | 394,33 | 20,90 |  | 394,00 | 21,45 |
| 027_1 | 527,00 | 452,00 | 28,21 |  | 515,00 | 27,69 |
| 027_2 | 498,00 | 444,00 | 27,37 |  | 500,00 | 27,00 |
| 027_3 | 532,00 | 456,00 | 27,28 |  | 476,00 | 25,66 |
| AVERAGE | 519,00 | 450,67 | 27,62 |  | 497,00 | 26,78 |
| 028_1 | 399,00 | 396,00 | 36,79 |  | 404,00 | 34,61 |
| 028_2 | 405,00 | 387,00 | 35,84 |  | 413,00 | 34,33 |
| 028_3 | 407,00 | 387,00 | 34,97 |  | 404,00 | 34,93 |
| AVERAGE | 403,67 | 390,00 | 35,87 |  | 407,00 | 34,62 |
| 029_1 | 896,00 | 810,00 | 36,02 |  | 835,00 | 37,01 |
| 029_2 | 898,00 | 824,00 | 38,45 |  | 870,00 | 37,29 |
| 029_3 | 861,00 | 773,00 | 37,28 |  | 834,00 | 37,30 |
| AVERAGE | 885,00 | 802,33 | 37,25 |  | 846,33 | 37,20 |
| 030_1 | 307,00 | 256,00 | 14,94 |  | 271,00 | 15,96 |
| 030_2 | 287,00 | 271,00 | 15,75 |  | 287,00 | 16,99 |
| 030_3 | 278,00 | 261,00 | 15,83 |  | 295,00 | 17,33 |
| AVERAGE | 290,67 | 262,67 | 15,51 |  | 284,33 | 16,76 |
| 031_1 | 275,00 | 251,00 | 27,33 |  | 337,00 | 28,69 |
| 031_2 | 311,00 | 269,00 | 27,50 |  | 347,00 | 29,56 |
| 031_3 | 314,00 | 273,00 | 28,43 |  | 348,00 | 29,87 |
| AVERAGE | 300,00 | 264,33 | 27,75 |  | 344,00 | 29,37 |
| 032_1 | 589,00 | 553,00 | 39,58 |  | 567,00 | 36,99 |
| 032_2 | 564,00 | 558,00 | 40,18 |  | 550,00 | 36,35 |
| 032_3 | 535,00 | 498,00 | 39,10 |  | 559,00 | 37,14 |
| AVERAGE | 562,67 | 536,33 | 39,62 |  | 558,67 | 36,83 |
| 033_1 | 562,00 | 552,00 | 24,70 |  | 600,00 | 25,18 |
| 033_2 | 533,00 | 568,00 | 24,33 |  | 638,00 | 25,49 |
| 033_3 | 525,00 | 560,00 | 24,47 |  | 620,00 | 24,08 |
| AVERAGE | 540,00 | 560,00 | 24,50 |  | 619,33 | 24,92 |
| 034_1 | 218,00 | 230,00 | 13,40 |  | 251,00 | 14,51 |
| 034_2 | 176,00 | 229,00 | 13,61 |  | 246,00 | 14,38 |
| 034_3 | 244,00 | 233,00 | 14,15 |  | 241,00 | 14,19 |
| AVERAGE | 212,67 | 230,67 | 13,72 |  | 246,00 | 14,36 |
| 035_1 | 461,00 | 435,00 | 23,51 |  | 480,00 | 24,59 |
| 035_2 | 459,00 | 442,00 | 25,21 |  | 469,00 | 25,07 |
| 035_3 | 427,00 | 406,00 | 23,10 |  | 490,00 | 25,05 |
| AVERAGE | 449,00 | 427,67 | 23,94 |  | 479,67 | 24,90 |
| 036_1 | 241,00 | 269,00 | 27,82 |  | 294,00 | 26,44 |
| 036_2 | 263,00 | 250,00 | 27,19 |  | 323,00 | 28,14 |
| 036_3 | 258,00 | 230,00 | 25,83 |  | 311,00 | 27,42 |
| AVERAGE | 254,00 | 249,67 | 26,95 |  | 309,33 | 27,33 |
| 037_1 | 116,00 | 112,00 | 33,65 |  | 123,00 | 28,00 |
| 037_2 | 135,00 | 112,00 | 34,06 |  | 128,00 | 30,74 |
| 037_3 | 113,00 | 96,00 | 32,00 |  | 136,00 | 30,97 |
| AVERAGE | 121,33 | 106,67 | 33,24 |  | 129,00 | 29,90 |
| 038_1 | 419,00 | 392,00 | 26,03 |  | 404,00 | 24,38 |
| 038_2 | 372,00 | 359,00 | 24,64 |  | 399,00 | 23,46 |
| 038_3 | 384,00 | 320,00 | 24,93 |  | 403,00 | 25,01 |
| AVERAGE | 391,67 | 357,00 | 25,20 |  | 402,00 | 24,28 |
| 039_1 | 199,00 | 214,00 | 13,24 |  | 223,00 | 13,09 |
| 039_2 | 146,00 | 212,00 | 13,58 |  | 217,00 | 12,35 |
| 039_3 | 239,00 | 217,00 | 13,68 |  | 229,00 | 13,24 |
| AVERAGE | 194,67 | 214,33 | 13,50 |  | 223,00 | 12,89 |
| 040_1 | 696,00 | 674,00 | 36,36 |  | 665,00 | 36,60 |
| 040_2 | 715,00 | 666,00 | 36,57 |  | 679,00 | 37,07 |
| 040_3 | 731,00 | 672,00 | 36,22 |  | 677,00 | 36,85 |
| AVERAGE | 714,00 | 670,67 | 36,38 |  | 673,67 | 36,84 |
| 041_1 | 717,00 | 680,00 | 33,91 |  | 699,00 | 32,43 |
| 041_2 | 727,00 | 652,00 | 33,39 |  | 646,00 | 32,12 |
| 041_3 | 711,00 | 684,00 | 33,75 |  | 666,00 | 31,74 |
| AVERAGE | 718,33 | 672,00 | 33,68 |  | 670,33 | 32,10 |
| 042_1 | 465,00 | 417,00 | 31,27 |  | 460,00 | 34,71 |
| 042_2 | 446,00 | 443,00 | 33,10 |  | 444,00 | 33,64 |
| 042_3 | 437,00 | 444,00 | 33,34 |  | 469,00 | 34,83 |
| AVERAGE | 449,33 | 434,67 | 32,57 |  | 457,67 | 34,39 |
| 043_1 | 511,00 | 482,00 | 31,79 |  | 472,00 | 32,15 |
| 043_2 | 532,00 | 474,00 | 31,42 |  | 479,00 | 32,60 |
| 043_3 | 456,00 | 469,00 | 30,97 |  | 502,00 | 32,79 |
| AVERAGE | 499,67 | 475,00 | 31,39 |  | 484,33 | 32,51 |
| 044_1 | 343,00 | 328,00 | 24,08 |  | 333,00 | 22,37 |
| 044_2 | 348,00 | 328,00 | 22,73 |  | 340,00 | 25,17 |
| 044_3 | 337,00 | 339,00 | 24,11 |  | 325,00 | 24,94 |
| AVERAGE | 342,67 | 331,67 | 23,64 |  | 332,67 | 24,16 |
| 045_1 | 630,00 | 599,00 | 29,48 |  | 589,00 | 31,14 |
| 045_2 | 633,00 | 605,00 | 28,82 |  | 571,00 | 30,97 |
| 045_3 | 606,00 | 571,00 | 28,48 |  | 596,00 | 29,94 |
| AVERAGE | 623,00 | 591,67 | 28,93 |  | 585,33 | 30,68 |
| 046_1 | 566,00 | 529,00 | 17,59 |  | 550,00 | 19,26 |
| 046_2 | 582,00 | 536,00 | 18,57 |  | 520,00 | 19,30 |
| 046_3 | 553,00 | 434,00 | 18,45 |  | 567,00 | 19,32 |
| AVERAGE | 567,00 | 499,67 | 18,20 |  | 545,67 | 19,29 |
| 047_1 | 718,00 | 689,00 | 32,62 |  | 670,00 | 32,06 |
| 047_2 | 733,00 | 686,00 | 31,73 |  | 644,00 | 31,53 |
| 047_3 | 697,00 | 597,00 | 31,30 |  | 682,00 | 31,90 |
| AVERAGE | 716,00 | 657,33 | 31,88 |  | 665,33 | 31,83 |
| 048_1 | 752,00 | 641,00 | 34,64 |  | 684,00 | 35,38 |
| 048_2 | 695,00 | 679,00 | 34,77 |  | 716,00 | 37,43 |
| 048_3 | 723,00 | 655,00 | 35,55 |  | 682,00 | 35,45 |
| AVERAGE | 723,33 | 658,33 | 34,99 |  | 694,00 | 36,09 |
| 049_1 | 571,00 | 501,00 | 36,15 |  | 507,00 | 34,22 |
| 049_2 | 575,00 | 536,00 | 34,84 |  | 547,00 | 36,01 |
| 049_3 | 571,00 | 554,00 | 36,43 |  | 517,00 | 33,03 |
| AVERAGE | 572,33 | 530,33 | 35,81 |  | 523,67 | 34,42 |
| 050_1 | 254,00 | 258,00 | 21,75 |  | 246,00 | 20,76 |
| 050_2 | 257,00 | 248,00 | 20,63 |  | 256,00 | 21,14 |
| 050_3 | 262,00 | 240,00 | 20,45 |  | 248,00 | 19,82 |
| AVERAGE | 257,67 | 248,67 | 20,94 |  | 250,00 | 20,57 |
| 051_1 | 307,00 | 299,00 | 14,60 |  | 288,00 | 13,55 |
| 051_2 | 287,00 | 269,00 | 13,70 |  | 285,00 | 14,01 |
| 051_3 | 274,00 | 282,00 | 13,87 |  | 289,00 | 13,84 |
| AVERAGE | 289,33 | 283,33 | 14,06 |  | 287,33 | 13,80 |
| 052_1 | 575,00 | 529,00 | 29,09 |  | 522,00 | 29,61 |
| 052_2 | 556,00 | 520,00 | 28,97 |  | 539,00 | 29,99 |
| 052_3 | 568,00 | 536,00 | 29,47 |  | 560,00 | 30,49 |
| AVERAGE | 566,33 | 528,33 | 29,18 |  | 540,33 | 30,03 |
| 053_1 | 559,00 | 524,00 | 33,10 |  | 539,00 | 34,75 |
| 053_2 | 572,00 | 533,00 | 34,08 |  | 555,00 | 33,32 |
| 053_3 | 521,00 | 521,00 | 33,45 |  | 526,00 | 34,14 |
| AVERAGE | 550,67 | 526,00 | 33,54 |  | 540,00 | 34,07 |
| 054_1 | 616,00 | 571,00 | 33,11 |  | 622,00 | 34,30 |
| 054_2 | 635,00 | 562,00 | 33,61 |  | 625,00 | 33,52 |
| 054_3 | 540,00 | 591,00 | 34,81 |  | 600,00 | 33,42 |
| AVERAGE | 597,00 | 574,67 | 33,84 |  | 615,67 | 33,75 |
| 055_1 | 557,00 | 495,00 | 38,25 |  | 532,00 | 37,72 |
| 055_2 | 520,00 | 547,00 | 39,72 |  | 495,00 | 39,97 |
| 055_3 | 545,00 | 540,00 | 37,69 |  | 479,00 | 38,40 |
| AVERAGE | 540,67 | 527,33 | 38,55 |  | 502,00 | 38,70 |
| 056_1 | 737,00 | 719,00 | 39,56 |  | 708,00 | 39,21 |
| 056_2 | 748,00 | 639,00 | 39,58 |  | 716,00 | 40,21 |
| 056_3 | 720,00 | 701,00 | 40,94 |  | 688,00 | 39,81 |
| AVERAGE | 735,00 | 686,33 | 40,03 |  | 704,00 | 39,74 |
| 057_1 | 1112,00 | 1086,00 | 36,10 |  | 1032,00 | 35,86 |
| 057_2 | 1135,00 | 994,00 | 33,63 |  | 1071,00 | 36,02 |
| 057_3 | 1083,00 | 1060,00 | 36,12 |  | 1069,00 | 35,72 |
| AVERAGE | 1110,00 | 1046,67 | 35,28 |  | 1057,33 | 35,87 |
| 058_1 | 593,00 | 491,00 | 24,83 |  | 518,00 | 26,44 |
| 058_2 | 563,00 | 523,00 | 25,93 |  | 550,00 | 26,12 |
| 058_3 | 542,00 | 515,00 | 25,24 |  | 521,00 | 26,95 |
| AVERAGE | 566,00 | 509,67 | 25,33 |  | 529,67 | 26,50 |
| 059_1 | 199,00 | 199,00 | 23,61 |  | 223,00 | 27,08 |
| 059_2 | 177,00 | 197,00 | 23,32 |  | 204,00 | 25,65 |
| 059_3 | 200,00 | 169,00 | 22,42 |  | 175,00 | 26,74 |
| AVERAGE | 192,00 | 188,33 | 23,12 |  | 200,67 | 26,49 |
