# Supplementary material for: Reliable and Accurate CD4+ T Cell Count and Percent by the Portable Flow Cytometer CyFlow MiniPOC and “CD4 Easy Count Kit-Dry”, as Revealed by the Comparison with the Gold Standard Dual Platform Technology
Source: PLoS One. 2015 Jan 26;10(1):e0116848. doi: 10.1371/journal.pone.0116848 (PMC4306486; doi:10.1371/journal.pone.0116848)
Supplement: S2 Table — Individual data points used for Figs. 4 and 5. (DOCX) [file pone.0116848.s004.docx]

**Table S2: Comparison between CyFlow MiniPOC and BC FC 500.**

|  | **BD FC 500** |  | **CyFlow® MiniPOC** |  |
| --- | --- | --- | --- | --- |
|  | **Dual Platform reference system** | | **Partec miniPOC CD4% count kit - dry** | |
| **ID sample** | **CD4 + T cells/ul** | **CD4%** | **CD4 + T cells/ul** | **CD4%** |
| 001 | 552 | 45 | 557 | 42 |
| 002 | 790 | 33 | 697 | 33 |
| 003 | 428 | 42 | 402 | 38 |
| 004 | 612 | 39 | 559 | 39 |
| 005 | 540 | 17 | 553 | 17 |
| 006 | 506 | 30 | 457 | 28 |
| 007 | 646 | 33 | 530 | 31 |
| 008 | 213 | 21 | 182 | 18 |
| 009 | 613 | 38 | 568 | 36 |
| 010 | 323 | 27 | 309 | 25 |
| 011 | 806 | 24 | 723 | 23 |
| 012 | 819 | 43 | 694 | 41 |
| 013 | 396 | 8 | 342 | 8 |
| 014 | 790 | 21 | 742 | 20 |
| 015 | 252 | 21 | 228 | 20 |
| 016 | 507 | 32 | 444 | 28 |
| 017 | 591 | 27 | 480 | 27 |
| 018 | 34 | 3 | 40 | 4 |
| 019 | 792 | 48 | 719 | 45 |
| 020 | 809 | 34 | 758 | 30 |
| 021 | 907 | 42 | 831 | 42 |
| 022 | 547 | 34 | 503 | 32 |
| 023 | 996 | 41 | 845 | 39 |
| 024 | 595 | 40 | 576 | 35 |
| 025 | 390 | 34 | 342 | 29 |
| 026 | 427 | 20 | 394 | 21 |
| 027 | 541 | 27 | 497 | 27 |
| 028 | 510 | 39 | 407 | 35 |
| 029 | 871 | 40 | 846 | 37 |
| 030 | 349 | 18 | 284 | 17 |
| 031 | 351 | 29 | 344 | 29 |
| 032 | 596 | 41 | 559 | 37 |
| 033 | 649 | 25 | 619 | 25 |
| 034 | 263 | 15 | 246 | 14 |
| 035 | 556 | 27 | 480 | 25 |
| 036 | 293 | 25 | 309 | 27 |
| 037 | 185 | 45 | 129 | 30 |
| 038 | 482 | 27 | 402 | 24 |
| 039 | 254 | 14 | 223 | 13 |
| 040 | 716 | 40 | 674 | 37 |
| 041 | 808 | 36 | 670 | 32 |
| 043 | 596 | 32 | 484 | 33 |
| 044 | 392 | 25 | 333 | 24 |
| 045 | 734 | 32 | 585 | 31 |
| 046 | 604 | 19 | 546 | 19 |
| 047 | 623 | 33 | 665 | 32 |
| 048 | 692 | 37 | 694 | 36 |
| 049 | 599 | 38 | 524 | 34 |
| 050 | 249 | 23 | 250 | 21 |
| 051 | 338 | 16 | 287 | 14 |
| 052 | 581 | 32 | 540 | 30 |
| 053 | 649 | 33 | 540 | 34 |
| 054 | 675 | 34 | 616 | 34 |
| 055 | 573 | 44 | 502 | 39 |
| 056 | 912 | 42 | 704 | 40 |
| 057 | 1115 | 37 | 1057 | 36 |
| 058 | 567 | 28 | 530 | 27 |
| 059 | 224 | 27 | 201 | 26 |
