# Supplementary material for: Reliable and Accurate CD4+ T Cell Count and Percent by the Portable Flow Cytometer CyFlow MiniPOC and “CD4 Easy Count Kit-Dry”, as Revealed by the Comparison with the Gold Standard Dual Platform Technology
Source: PLoS One. 2015 Jan 26;10(1):e0116848. doi: 10.1371/journal.pone.0116848 (PMC4306486; doi:10.1371/journal.pone.0116848)
Supplement: S3 Table — Individual data points used for Fig. 6. (DOCX) [file pone.0116848.s005.docx]

**Table S3: Precision assessment.**

**A:** CD4+ T cells/µl

| **sample** | **single** | **bulk** |
| --- | --- | --- |
| 1 | 202,00 | 189,00 |
| 2 | 185,00 | 189,00 |
| 3 | 198,00 | 188,00 |
| 4 | 182,00 | 188,00 |
| 5 | 209,00 | 186,00 |
| 6 | 190,00 | 186,00 |
| 7 | 195,00 | 184,00 |
| 8 | 198,00 | 185,00 |
| 9 | 186,00 | 184,00 |
| 10 | 188,00 | 192,00 |
| Mean | 193,30 | 187,10 |
| SD | 8,55 | 2,56 |
| SD % | 4,42 | 1,37 |

**B:** CD4+ %

| **sample** | **single** | **bulk** |
| --- | --- | --- |
| 1 | 19,98 | 18,39 |
| 2 | 18,42 | 17,72 |
| 3 | 19,65 | 17,48 |
| 4 | 19,24 | 17,51 |
| 5 | 19,19 | 17,48 |
| 6 | 19,97 | 18,39 |
| 7 | 19,25 | 17,54 |
| 8 | 18,60 | 18,01 |
| 9 | 20,05 | 18,91 |
| 10 | 17,52 | 18,16 |
| Mean | 19,19 | 17,96 |
| SD | 0,81 | 0,50 |
| SD % | 4,23 | 2,76 |
