# Supplementary material for: Reliable and Accurate CD4+ T Cell Count and Percent by the Portable Flow Cytometer CyFlow MiniPOC and “CD4 Easy Count Kit-Dry”, as Revealed by the Comparison with the Gold Standard Dual Platform Technology
Source: PLoS One. 2015 Jan 26;10(1):e0116848. doi: 10.1371/journal.pone.0116848 (PMC4306486; doi:10.1371/journal.pone.0116848)
Supplement: S4 Table — Individual data points used for Fig. 7. (DOCX) [file pone.0116848.s006.docx]

**Table S4: Carry-over contamination analysis.**

**A:** CD4+ T cells/µl

|  | analysis 1 | analysis 2 | analysis 3 | mean | sd | sd% |
| --- | --- | --- | --- | --- | --- | --- |
| medium | 521.00 | 508.00 | 480.00 | 503.00 | 20.95 | 4.17 |
| high | 861.00 | 829.00 | 826.00 | 838.67 | 19.40 | 2.31 |
| low | 42.00 | 41.00 | 41.00 | 41.33 | 0.58 | 1.40 |
| medium | 502.00 | 506.00 | 509.00 | 505.67 | 3.51 | 0.69 |
| medium | 502.00 | 489.00 | 507.00 | 499.33 | 9.29 | 1.86 |
| low | 41.00 | 41.00 | 40.00 | 40.67 | 0.58 | 1.42 |
| low | 44.00 | 40.00 | 43.00 | 42.33 | 2.08 | 4.92 |
| high | 773.00 | 808.00 | 803.00 | 794.67 | 18.93 | 2.38 |
| high | 826.00 | 808.00 | 807.00 | 813.67 | 10.69 | 1.31 |
| medium | 495.00 | 500.00 | 541.00 | 512.00 | 25.24 | 4.93 |

**B:** CD4+ %

|  | analysis 1 | analysis 2 | analysis 3 | mean | sd | sd% |
| --- | --- | --- | --- | --- | --- | --- |
| medium | 27.83 | 27.61 | 29.83 | 28.42 | 1.22 | 4.30 |
| high | 40.29 | 41.21 | 41.94 | 41.15 | 0.83 | 2.01 |
| low | 3.68 | 4.03 | 4.19 | 3.97 | 0.26 | 6.58 |
| medium | 28.07 | 31.91 | 32.10 | 30.69 | 2.27 | 7.41 |
| medium | 28.14 | 29.54 | 32.48 | 30.05 | 2.22 | 7.37 |
| low | 4.00 | 4.05 | 4.19 | 4.08 | 0.10 | 2.41 |
| low | 4.00 | 3.67 | 4.25 | 3.97 | 0.29 | 7.32 |
| high | 41.52 | 41.12 | 40.69 | 41.11 | 0.42 | 1.01 |
| high | 41.30 | 39.45 | 42.38 | 41.04 | 1.48 | 3.61 |
| medium | 29.64 | 29.44 | 29.45 | 29.51 | 0.11 | 0.38 |
