# Supplementary material for: Reliable and Accurate CD4+ T Cell Count and Percent by the Portable Flow Cytometer CyFlow MiniPOC and “CD4 Easy Count Kit-Dry”, as Revealed by the Comparison with the Gold Standard Dual Platform Technology
Source: PLoS One. 2015 Jan 26;10(1):e0116848. doi: 10.1371/journal.pone.0116848 (PMC4306486; doi:10.1371/journal.pone.0116848)
Supplement: S2 Document — (PDF) [file pone.0116848.s002.pdf]

sysmex

Sysmex Partec GmbH · Am Flugplatz 13 · 02828 Görlitz · Germany

Sysmex Partec GmbH
Am Flugplatz 13
02828 Görlitz, Germany
Phone +49 3581 8746-0
Fax +49 3581 8746-70
info@sysmex-partec.com

To whom it may concern

Münster, 26/11/2014

Dear Editor

I hereby, Francesco Marinucci, Director of the Essential Healthcare division at Sysmex Partec GmbH, authorize the publication of the picture of the instrument CyFlow miniPOC as part of the paper D-14-38679R1 recently submitted by Prof. Cossarizza and colleagues to your journal.

Kind regards,

Dr. Francesco Marinucci, PhD, EurClinChem
Essential Healthcare, Director
Sysmex Partec GmbH

sysmex
Sysmex Partec GmbH
Am Flugplatz 13
02828 Görlitz
Germany
